# Development and Validation of Open-Source Activity Intensity Count and Activity Intensity Classification Algorithms from Raw Acceleration Signals of Wearable Sensors

**DOI:** 10.3390/s20236767

**Published:** 2020-11-26

**Authors:** Isabelle Poitras, Jade Clouâtre, Laurent J. Bouyer, François Routhier, Catherine Mercier, Alexandre Campeau-Lecours

**Affiliations:** 1 Centre for Interdisciplinary Research in Rehabilitation and Social Integration, Quebec City, QC G1M 2S8, Canada; isabelle.poitras.2@ulaval.ca (I.P.); jade.clouatre.1@ulaval.ca (J.C.); Laurent.Bouyer@rea.ulaval.ca (L.J.B.); francois.routhier@rea.ulaval.ca (F.R.); catherine.mercier@rea.ulaval.ca (C.M.); 2 Department of Rehabilitation, Laval University, Quebec City, QC G1V 0A6, Canada; 3 Department of Mechanical Engineering, Laval University, Quebec City, QC G1V 0A6, Canada

**Keywords:** wearable sensors, activity level quantification, activity level classification, rehabilitation technologies, rehabilitation engineering, accelerometers, physical activity quantification

## Abstract

Background: A popular outcome in rehabilitation studies is the activity intensity count, which is typically measured from commercially available accelerometers. However, the algorithms are not openly available, which impairs long-term follow-ups and restricts the potential to adapt the algorithms for pathological populations. The objectives of this research are to design and validate open-source algorithms for activity intensity quantification and classification. Methods: Two versions of a quantification algorithm are proposed (fixed [FB] and modifiable bandwidth [MB]) along with two versions of a classification algorithm (discrete [DM] vs. continuous methods [CM]). The results of these algorithms were compared to those of a commercial activity intensity count solution (ActiLife) with datasets from four activities (*n* = 24 participants). Results: The FB and MB algorithms gave similar results as ActiLife (r > 0.96). The DM algorithm is similar to a ActiLife (r ≥ 0.99). The CM algorithm differs (r ≥ 0.89) but is more precise. Conclusion: The combination of the FB algorithm with the DM results is a solution close to that of ActiLife. However, the MB version remains valid while being more adaptable, and the CM is more precise. This paper proposes an open-source alternative for rehabilitation that is compatible with several wearable devices and not dependent on manufacturer commercial decisions.

## 1. Introduction

The popularity of wearable devices to monitor physical activity (PA) has increased widely during the last decades. Plenty of wearable devices, from step counters to sleep monitors, are now available to provide a broad spectrum of physiological measurements and feedbacks (e.g., step count [1], level of activity [2], upper limb activity count [3], distance traveled [4], heart rate history [5]). These devices have been widely adopted for everyday monitoring of PA, such as personal training (by providing to the user a real-time feedback of their performance [6]), but are yet to be implemented in clinical physical therapy. From a clinical perspective, healthcare services could benefit from wearable monitoring devices. Indeed, there is a vast diversity of available miniature sensors (inertial measurement units, electromyography, heart rate sensors, dermatological sensors [sweating], etc.) and physical outcomes that can be extracted from such sensors. Combined with the possibility to automatically save data on a secure cloud service (as with most commercial wearable sensors), it would be possible to give direct feedback to patients or to give better insight to clinicians about their patients’ everyday activity evolution, allowing them to provide feedback and enabling more personalized interventions. For example, stroke survivors and their rehabilitation professionals could benefit from a more comprehensive and quantitative assessment of the real use of the affected arm after returning home by receiving daily feedback [7]. Children with cerebral palsy would also benefit from a closer monitoring and feedback on their capacities before beginning school to maximize functional gains [3]. Injured workers could benefit from a more realistic and quantitative assessment [8] of environmental risk factors, which would help them return to work more quickly and safely. Wearable sensors have the potential to improve clinical rehabilitation monitoring and efficiency by allowing an objective, non-invasive and inexpensive way to evaluate physical functions [9].

However, wearable sensors are not commonly used in clinical practice as of now. The challenge does not simply reside in the development of sensors and data processing algorithms, but also in the transformation from raw sensor data to a feedback that is clinically meaningful to end-users. Such a transformation crucially requires interdisciplinary collaboration. Furthermore, a major issue of available commercial wearable devices is that the underlying algorithms are often protected either by intellectual property or trade secrets [10]. This leads to two problems: (1) the algorithms are not openly available which impairs data reproducibility and long-terms follow-ups if the technology becomes discontinued; and (2) the algorithms might be based on data from young and healthy subjects, thus reducing the variability of movement patterns and restricting their readiness to be used in pathological population (e.g., children living with cerebral palsy or older stroke survivors). Reduction in movement speed (e.g., multiple sclerosis) [11], impossibility to maintain or perform a calibration position (e.g., spasticity secondary to an upper motoneuron injury) [12], tremors (e.g., Parkinson disease) [13] or muscle spasms (e.g., spinal cord injury) [14] are common problems that are encountered and that must be considered when using portable devices for rehabilitation or medical purposes requiring algorithm optimization. This uncertainty about the sustainability of commercial devices over time and the lack of flexibility to adapt the algorithms to different clinical populations are major concerns for researchers and clinicians.

A popular outcome that has been used in many rehabilitation studies [15,16,17,18] is the activity intensity count to provide an insight of the physical condition/participation of their patient to the clinician. This variable is extracted from accelerometers and consists of a scaled quantity of activity performed during a certain amount of time. However, as is generally the case, algorithms from commercial activity intensity count wearable devices are not publicly available (or open source) since they are protected by trade secret. The development of an open-source activity intensity count algorithm is necessary to ensure the sustainability of data processing and to enable researchers to optimize the algorithm to given clinical populations. To this end, BrØnd & al. [10] have developed an open-source algorithm statistically comparable to commercial activity intensity count solutions (e.g., ActiGraph GT9X, ActiLife). Interestingly, the algorithm can be used with other accelerometer sensors or inertial measurement units (IMU) (which comprise accelerometers), as they are based on raw data acceleration (in the case where the commercial technology would be discontinued), assuming that the accelerometers’ specifications are similar (raw data between accelerometers is typically similar but the accelerometer range must also be similar).

However, the proposed algorithm in BrØnd & al. [14] has two main issues for clinical applications. First, from its design, the algorithm obligatorily requires one to downsample the acceleration signal sampling rate to 30 Hz, which can be too low for applications such as running (where a sampling rate of at least 250 Hz is recommended [19]). Indeed, high movement speeds require a higher sampling rate to ensure high data quality acquisitions and reduce the risk of missing data [20]. Sports medicine in general (e.g., swimming, throwing a ball) and working environments (e.g., industrial factory work) with high biomechanical requirements are other examples of applications requiring high sampling rates [21]. Secondly, the band-pass filter parameters of the proposed algorithm in BrØnd & al. [10] cannot be modified because of the numerical optimization process used to find the parameters (the filter parameters are complicated and they cannot be changed to accommodate another band-pass frequency, for instance). Thus, the filter cannot be modified to adapt to various pathological conditions and this could lead to underestimated or overestimated activity quantification depending on the clinical population assessed. For instance, adapting the filter parameters (band-pass cut-off frequency, notch filter) could help reduce the impact of tremors on the quantification of activity intensity count data (e.g., tremors in Parkinson’s patients do not count as activity count), or involuntary movements (e.g., spasms within children living with cerebral palsy) or very slow movements.

The objectives of the current study are to design and validate two activity quantification algorithms and an activity intensity classifier: (1) a fixed bandwidth algorithm, which replicates commercial activity intensity counts as closely as possible, allowing acceleration signal processing regardless of sampling rates; (2) a modifiable bandwidth algorithm that allows us to easily adapt the filter parameters, regardless of sampling rates; and (3) an activity intensity classification algorithm that allows the modification of cut-off values according to the studied population, thereby reducing potential saturation effects.

The paper is structured as follows: the descriptions of the two activity quantification algorithms are first presented, followed by the description of the activity intensity classification algorithm. Thereafter, the protocols used to validate the proposed algorithms are compared to commercial solutions. Then, the results are presented, followed by a discussion and conclusion.

The proposed open-source software is available at https://github.com/ingreadaptulaval/activitycounts and provides the quantification algorithms along with cut-points.

## 2. Materials and Methods

In this section, the proposed activity quantification algorithms (fixed and modifiable bandwidths) and the activity intensity classification scale are presented, along with the protocol used to validate them against commercial solutions.

### 2.1. Activity Quantification Algorithm

Two algorithms are proposed: a fixed bandwidth algorithm and a modifiable bandwidth algorithm (which offers more flexibility).

**Figure 1:**
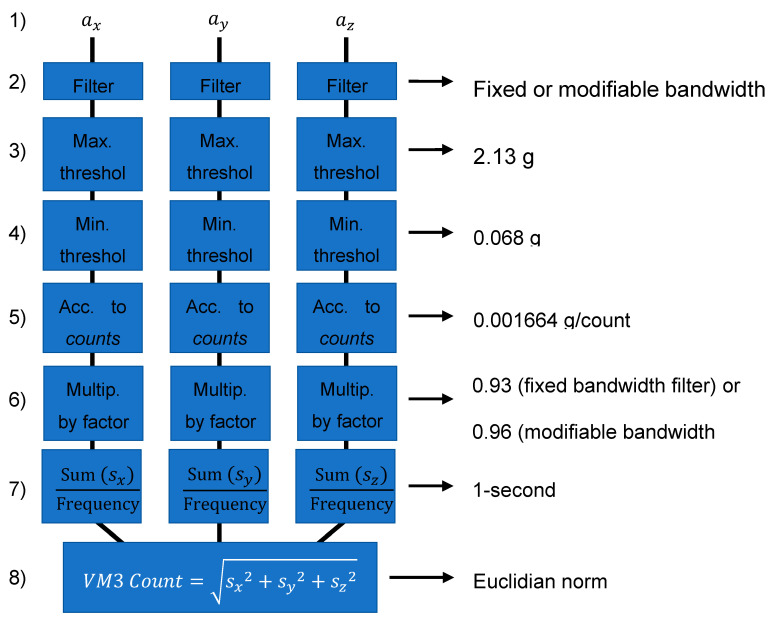
Activity quantification algorithm scheme.

Both algorithms have the same basic structure, which is first detailed here (see eight steps below) and summarized in Figure 1.

(1) The 3D linear acceleration signals (ax, ay and az) are first sampled at a fixed frequency (e.g., 30 Hz or 100 Hz).

(2) Each of the three signals is filtered with a passband filter (specific to each algorithm and described below).

(3) A saturation (maximum threshold) limit of 20.874 m/s^2^ (2.13 g with g = 9.81 m/s^2^) [10] is applied to each signal.

(4) A deadband (minimum threshold) of 0.6664 m/s^2^ (i.e., 0.068 g) [10] is applied to each signal.

(5) A scaling is then applied to convert the acceleration signal into a metric referred to as activity counts. The scaling is 1 count = 0.001664 g = 0.0163072 m/s^2^ [22]. This scaling could be omitted but is applied to match with the “count” definition from ActiGraph which was defined as such for historical reasons [23].

(6) The counts are then multiplied by a factor (0.93 for the fixed bandwidth filter and 0.96 for the modifiable bandwidth filter), as in BrØnd & al. [10], to better match the commercial activity counts results. These values (0.93 and 0.96) were optimized with the least squares method to best match ActiLife results.

(7) Each of the three resulting signals from steps 1 to 5 are converted into 1-s epochs. This consists of the sum of the counts acquired over 1 s divided by the sampling frequency to obtain the mean activity count/sec, defined as $s_{x_{i}}$, $s_{y_{i}}$ and $s_{z_{i}}$, where i is the epoch number.

(8) For each epoch, the total tri-axial vector magnitude (VM3) count is obtained with a Euclidian norm:

(1) $$VM3\;Count_{i}=\sqrt{s_{x_{i}}{}^{2}+s_{y_{i}}{}^{2}+s_{z_{i}}{}^{2}}$$

#### 2.1.1. Fixed Bandwidth Algorithm

**Figure 2:**
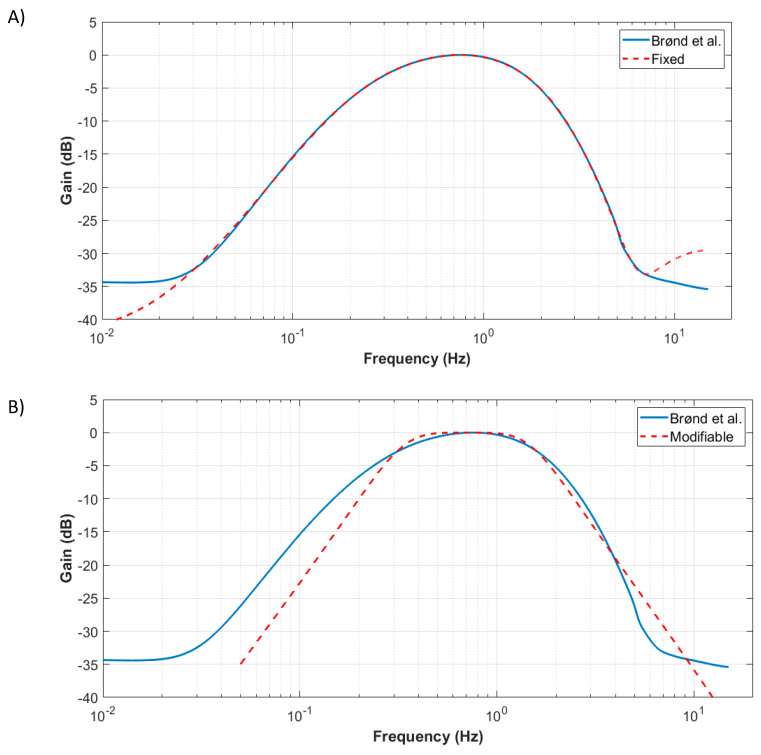
Bode diagrams of (**A**) the fixed bandwidth filter and (**B**) the modifiable bandwidth filter. The BrØnd et al. filter is from [10].

The fixed bandwidth algorithm exactly follows the general sequence presented in the previous section. The focus in this subsection is on the bandpass filter applied at step 2 of the algorithm. In BØnd & al. [10], the commercial bandpass filter characteristics were identified by generating discrete sinewave signals and successively sending them to the software. A discrete bandpass transfer function (at 30 Hz) of order 20 was optimized to represent the commercial activity counts filter. However, being defined only at 30 Hz precludes the algorithm use at other sampling frequencies, which is vital for other applications (e.g., running, requiring sampling rates of 100 Hz to 500 Hz). Furthermore, it was found that the high order of the filter also prevented the conversion of the discrete transfer function to other sampling frequencies due to numerical instability. In the framework of this paper, a continuous 8th-order bandpass transfer function has thus been used to mimic the commercial activity count bandpass frequency response. Using MATLAB, a genetic optimization algorithm was used, starting from random continuous filter parameters, and discretized to a 30 Hz discrete bandpass filter at each optimization step to match the filter from BØnd & al. [10]. As the optimization result is the continuous filter parameter, it is then possible to discretize the filter to any sampling frequency (e.g., 100 Hz). Figure 2A compares the proposed filter to that of BØnd & al. [10].

#### 2.1.2. Modifiable Bandwidth Algorithm

This section proposes a modifiable bandpass filter version of the fixed bandwidth algorithm. The advantage of the fixed bandwidth algorithm presented in the previous section is that the resulting activity intensity counts are closer to commercial activity count solutions than to the modifiable bandwidth algorithm. This might be an advantage because commercial activity count solutions have been validated in the literature, and the results can be compared to those of previous studies that used commercial solutions. Indeed, the commercial solutions’ passband filters were designed for healthy subjects and might not be optimal for other clinical populations. However, because the fixed passband 8th-order filter was obtained through an optimization process to best represent the commercial solution, changing its bandwidth is not an obvious procedure. A simpler 4th-order passband Butterworth filter is thus proposed. It was designed using MATLAB with the “butter” function and then discretized to the required sampling frequency (e.g., 30 Hz or 100 Hz). The default filter parameters (i.e., high and low cutoff frequencies) were obtained to be as close as possible to the BrØnd & al. [10] filter using a least square optimization approach and are 0.305 Hz and 1.615 Hz, respectively. Figure 2B presents the proposed modifiable filter compared to BrØnd & al. [10]. With this filter, the bandwidth can now be easily modified (by changing the high and low cutoff frequencies) to adapt to the needs of different activities/clinical populations. One should note that other algorithm parameters (deadband and threshold) can be adapted as well, as for the fixed bandwidth algorithm. However, in exchange for such modularity, the modifiable bandwidth leads to larger deviations from commercial activity counts as more approximations are required.

### 2.2. Activity Intensity Classification

Based on the activity intensity counts, two activity intensity classification algorithms are proposed. The goal of these algorithms is to determine the proportion of time spent in different categories of activity intensity during an activity such as “light, moderate, vigorous, very vigorous”. The number of categories and the threshold for each class (referred to as cutpoints) can vary depending on the activity. Therefore, in order to compare the proposed algorithm to commercial activity intensity count solutions, the Freedson Adult VM3 scale [24] was used. It is divided into four activity intensities (light, moderate, vigorous and very vigorous) corresponding to the different ranges of activity counts per minute that are shown in Table 1. The number of categories and cutpoint levels can easily be changed to any other value as needed.

Two versions of the activity intensity classification algorithms are proposed: one referred to as the discrete classification method and the other one as the continuous classification method.

#### 2.2.1. Discrete Classification Method

The discrete classification method consists of summing counts (which, based on the quantification algorithm are in 1-s epochs) into 1-min-epochs. A 3-min test therefore has three epochs: the sum of the activity level counts from 0 to 60 s, from 60 to 120 s and from 120 to 180 s. Then, each epoch is associated with an activity intensity category based on the threshold values presented in Table 1 (e.g., light). Finally, the algorithm outputs the number of 1-min epochs for each category along with the representation percentage in each activity intensity class. This discrete classification method is the one used in commercial solutions such as ActiLife.

#### 2.2.2. Continuous Classification Method

One potential problem with the discrete classification method is that the results may greatly vary depending on how the activity levels fall within the 1-min epochs.

**Figure 3:**
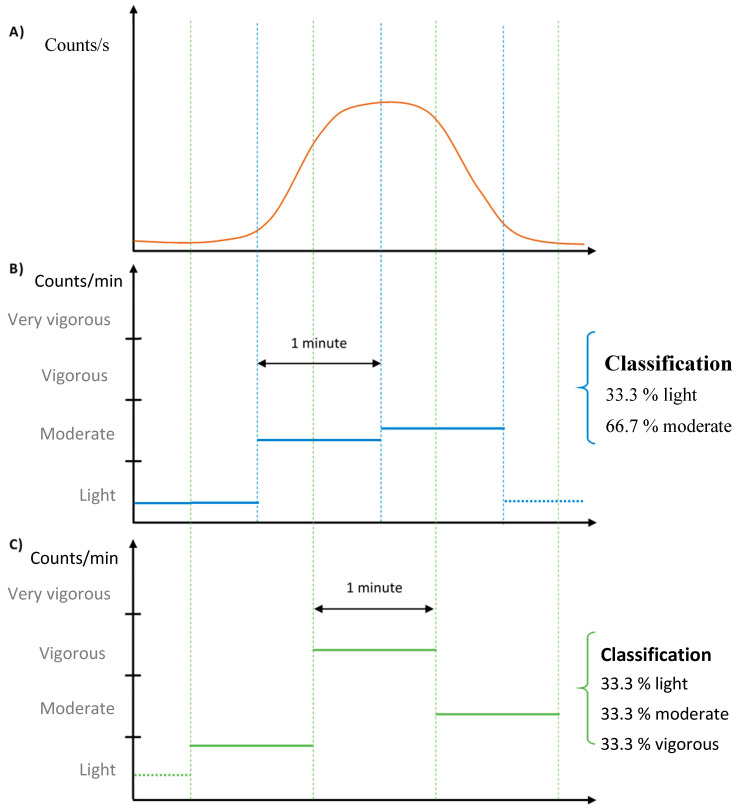
Representation of the impact of the discrete summation by ActiLife with a simple example of activity classification. (**A**) 1 s-epoch graph of an activity of approximately 3 min. (**B**) A first result possible for the summation of 1-min blocks for the activity in (**A**). (**C**) A second result possible for the summation of 1-min blocks for the activity in (**A**).

Figure 3 presents a fictive example demonstrating this limitation. Figure 3A shows an activity level curve that lasts three minutes. Figure 3B displays the resulting 1-min epoch categories, assuming that the activity started at the same time as data acquisition. Figure 3C shows the resulting 1-min epoch categories assuming that the activity started 30 s after the onset of data acquisition. As seen when comparing Figure 3B,C, the end result is quite different (the percentage in each class is different).

In order to alleviate this drawback, a new continuous classification algorithm is proposed, based on a sliding window approach. Rather than using back-to-back one minute-epochs, continuous one minute-epochs are used: at each second, a one minute-epoch is created by taking the 30 preceding and 30 following seconds. This means that for a 3-min test, 180 overlapping one minute-epochs are used and categorized. The aim of this algorithm is to be more precise in the cutpoint evaluation by alleviating the drawback of the discrete classification method where the one minute-epoch level results depend on when the movement starts with respect to data acquisition onset. Finally, the algorithm outputs the number of epochs for each category together with the representation percentage in each activity intensity class.

### 2.3. Experimental Procedure

Data were gathered from four different projects involving accelerometry measures. These projects were: (1) Activity of daily living (24-h data collection); (2) Bilateral manipulation in daily living tasks; (3) Manual wheelchair propulsion; and (4) Real-world working environment—Nordic expedition project on the Amundsen Research Ice breaker. In this section, the participants involved in each experiment and the experimental setup of each project are described.

#### 2.3.1. Participants and Experimental Setup

- Activities of daily living—24-h data collection (project 1):

Four participants (25 ± 0.82 years old, two men, four right-handed) wore two watches (one on each wrist, sampling rate = 100 Hz, ±8G, ActiGraph GT9X-BT, ActiGraph, LLC) for 24 h. Activities included but were not limited to running, walking, driving a car, dressing or working at a computer (see Annex 1, Table 1 for more details). Participants were instructed to fill in a custom-designed activity logbook reporting the activities they performed each hour, and to remove the device only for activities requiring water immersion (e.g., swimming, taking a shower). See Appendix A Table A1 for a synthesis of the activities reported. Recruitment criteria were (1) being aged 18 years or older, and (2) having no musculoskeletal, neurological disorders or pain. Every participant gave written informed consent prior to participation and the project was approved by the local ethics committee (CIUSSS-CN; project #2018-609).

- Bilateral manipulation in daily living tasks (project 2):

Ten participants (27.9 ± 7 years old, three men, nine right-handed) wore two watches (on each wrist [dominant and non-dominant hand], sampling rate = 100 Hz, ±8G, Actigraph GT9X-BT, ActiGraph, LLC) during eight specific activities of daily living. Activities: (1) washing a table, (2) making coffee, (3) setting the table, (4) serving a glass of water, (5) cutting therapeutic putty, (6) folding towels, (7) putting toothpaste on a toothbrush, (8) walking, performed in a standardized kitchen of a rehabilitation center. Tasks were selected to require the use of both upper limbs. Each task took between 1.5 and 2 min, for a total of approximately 15 min. Recruitment criteria were (1) being 18 years or older, (2) having no musculoskeletal, neurological disorder or pain that could interfere with the task. Participants were recruited using the research center and Laval University’s mailing lists. Each participant gave written informed consent prior to participation. The project was approved by the local ethics committee (CIUSSS-CN; project #2018-609).

- Manual wheelchair propulsion (project 3):

Seven manual wheelchair users (45.8 ± 17.2 years old, six women and one man wore a watch (sampling rate = 30 Hz, ±8G, Actigraph, GT3X-BT, ActiGraph, LLC) on their dominant wrist during two days at two different data collection times: before (T1) and after (T2) following a wheelchair training program. Participants had different diagnoses (multiple sclerosis [*n* = 1], spina bifida [*n* = 2], spinal cord injury [*n* = 1], cerebral palsy [*n* = 1], Friedreich ataxia [*n* = 1] and post-poliomyelitis syndrome [*n* = 1]) and they used their manual wheelchair for more than 5 h per day. All participants gave their informed written consent prior to participation and the project was approved by the local ethics committee (CIUSSS-CN; project #2016-493). Subject 2-T2 was excluded due to technical problems with data collection.

- Real-world working environment—Nordic expedition project on the Amundsen (project 4):

Two healthy workers (37 and 40 years old, two women, both right-handed) were recruited from a Nordic expedition project aboard an icebreaker (NGCC Amundsen, Canadian Coast Guard) (total data collection of six weeks). They were instructed to wear two watches (24/7) (sampling rate = 30 Hz, ±8G, Actigraph GT3X-BT, ActiGraph, LLC), one on the middle of the thigh and one fixed in a customized pair of shorts at thigh and L1-L5 levels (trunk and leg). They also filled a task logbook at the end of each workday. Recruitment criteria were (1) being 18 years or older, (2) being assigned to a >=6-week work shift on the icebreaker, (3) having no musculoskeletal, neurological disorders or pain that would limit their work capacity. Each participant gave written informed consent prior to the experiment and the project was approved by the local ethics committee (CIUSSS-CN; project #2017-539).

#### 2.3.2. Statistical Analysis

##### Activity Quantification Algorithm

Descriptive analyses (sum, mean and standard deviation [SD]) were used to describe the recording sessions (e.g., time and activity count) and participants (e.g., age). Pearson correlation coefficients were calculated between the data processed by ActiLife 6 (ActiGraph, LLC) and by both (fixed and modifiable) bandwidth algorithms. A Bland–Altman plot for each subject, time of measure (when applicable) and algorithm was used to investigate the validity under free living (outcomes: mean difference, number of data outside of 1.96SD, upper and lower limits of agreement). The level of significance for all analyses was set at *p* < 0.05. A linear regression was used to evaluate any association between error size of the difference between the proposed algorithm compared to the commercial solution and intensity of activity.

##### Activity Intensity Classification

Descriptive analyses (mean and SD) were used to describe the proportion of time spent at each activity level. Agreement between the classification of the five intensity categories was assessed using a correlation coefficient analysis (considering as very low [0.0 to 0.3], low [0.3 to 0.5], moderate [between 0.5 and 0.7], high [between 0.7 and 0.9] or very high correlation [>0.9] [25]). All statistical analyses and data processing were performed with a customized MATLAB program (R2018b, 64bit). A Spearman correlation coefficient was calculated for both algorithms comparing the commercially available one to the discrete and the continuous methods separately (significance level = *p* < 0.05). A NparLD analysis was performed to compare the commercial algorithm to the discrete and continuous methods (2 factors; algorithm tested and level of physical activity). NparLD is a robust method that does not require a normal distribution and homoscedasticity while allowing us to assess datasets with repeated measures [26]. Post-hoc analyzes compared commercial to discrete methods and commercial to continuous methods for each level of PA. Statistical significance was set at *p* < 0.05 for the main and interaction effects and at *p* < 0.00625 for the post-hoc analyses (Bonferroni correction).

## 3. Results

### 3.1. Activity Quantification Algorithm—Fixed bandwidth algorithm

**Figure 4:**
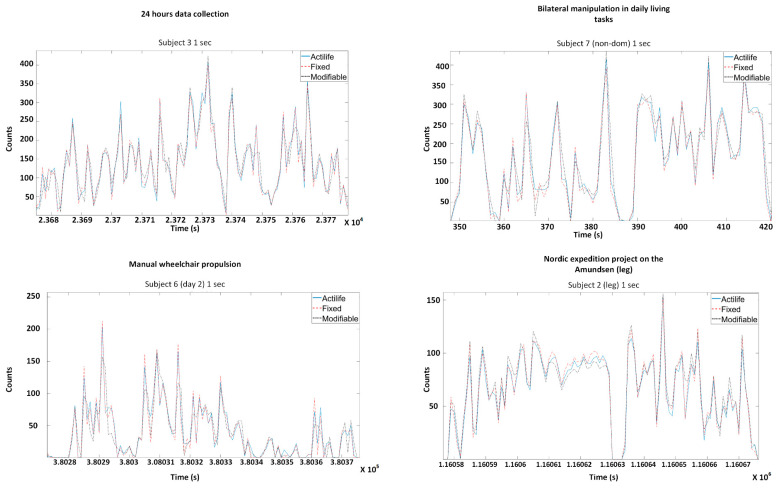
Representative subjects for each project comparing the commercial activity count to the fixed and modifiable algorithm.

Table 2 presents the outcomes of the fixed bandwidth algorithm for each project. For each dataset, the total activity count was averaged to counts per minutes and ranged from 90.2 to 7912.1 counts with a grand average of 54.7 ± 67.4 counts. The relative differences between the commercial activity and fixed bandwidth counts were ≤2.1% for all projects (smallest for project 3 with a mean of 0.3%). The average Pearson correlation coefficient was 0.99 ± 0.001 (all *p* < 0.001). Figure 4 shows a representative subject for each project at an epoch time of 1 s comparing activity counts for both algorithms showing a high level of similarity between the commercial solutions and the fixed bandwidth algorithms.

**Figure 5:**
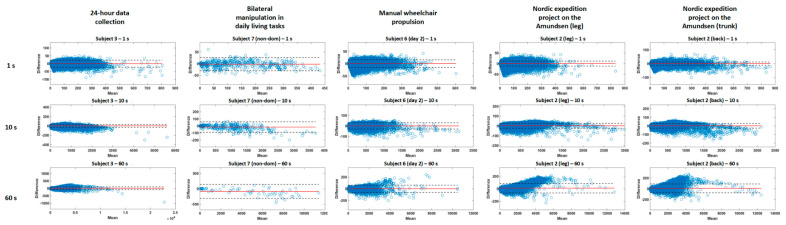
Bland–Altmann plot for representative subjects for each project and epochs of 1, 10 and 60 s for the fixed bandwidth algorithm.

The Bland–Altman analysis of the proposed fixed algorithm is presented in Figure 5, per project, for epochs of 1, 10, and 60 s for a representative subject. The absolute number of data points out of the limits of agreement ranged from 21 to 19,170 for epochs of 1 s, from 1 to 5037 for epochs of 10 s and from 0 to 1357 for epochs of 60 s. This represents a relative error of 3.1% ± 0.9% for project 1, 4.4% ± 2.3% for project 2, 1.3% ± 0.8% for project 3 and 2.5% ± 1.3% for project 4, showing smaller error ranges for longer recording times (project 3 and 4) while still presenting high concurrent validity for shorter ones. The relative mean errors were 2.3% ± 1.6% for 1 s, 3.2% ± 1.8% for 30 s and 3.8% ± 3% for 60 s. The mean differences, limits of agreement (±1.96SD) and regression coefficients are presented in Appendix A Table A2 and Table 2. The regression coefficients ranged from 0.47 to 0.88, representing a moderate to high relationship between PA and absolute error (highest coefficients for 60 s epochs and longer recording times [project 3 and 4, mobility projects in manual wheelchair users and working context]).

### 3.2. Activity Quantification Algorithm—Modifiable Bandwidth Algorithm

Table 3 presents the outcomes of the modifiable bandwidth algorithm for each project. For each dataset, the total activity count was averaged to counts per minutes and ranged from 88.2 to 8271.7. The absolute difference between this algorithm output and the commercial solution ranged from −5.7 (project 3—subject 7) to 358.5 (project 2—subject 8), with a grand average of 71.7 ± 94.5 counts. The average relative differences between the commercial activity counts and fixed bandwidth counts were ≤4.1% for all projects (smallest for project 3 with a mean of 1.7%). Figure 4 shows a representative subject for each project with an epoch time of 1 s, showing a high level of similarity between the commercial solutions and the proposed algorithm.

**Figure 6:**
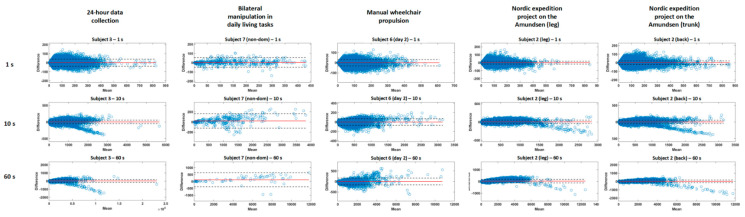
Bland–Altmann plot for a representative subject for each project at an epoch of 1 s, 10 s and 60 s for the modifiable bandwidth algorithm.

The Bland–Altman analysis of the proposed modifiable algorithm is presented in Figure 6, per project, for epochs of 1, 10 and 60 s for a representative subject. The absolute number of data points out of the limits of agreement ranged from 22 to 25,718 for epochs of 1 s, from 3 to 5241 for epochs of 10 s and from 0 to 1333 for epochs of 60 s. This represents a relative error of 3.3% ± 0.9% for project 1, 4.1% ± 2.3% for project 2, 1.3% ± 0.8% for project 3 and 1.5% ± 1.0% for project 4, showing smaller error ranges for longer recording times (project 3 and 4), while still presenting high concurrent validity for shorter ones. The relative mean errors were 2.5% ± 1.6% for 1 s, 3.8% ± 2.4% for 30 s and 2.4% ± 2.1% for 60 s. The mean differences, limits of agreement (±1.96SD) and regression coefficients are presented in Appendix A Table A2 and Table 3. The regression coefficients ranged from 0.31 to 0.90, representing a moderate to high relationship between PA and absolute error (highest coefficients of 60 s epochs and longer recording times [project 3 and 4, mobility projects in manual wheelchair users and working context]).

### 3.3. Activity Intensity Classification

**Figure 7:**
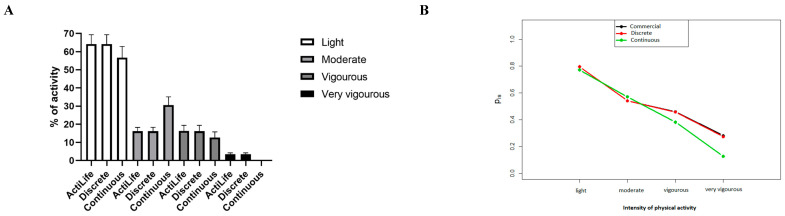
(**A**): Average and standard error of the mean of activity count classification results for the commercial activity count, the discrete and the continuous methods; (**B**): Relative effects of the three algorithms (commercial, discrete and continuous methods).

An overview of the activity intensity classification algorithm results is presented in Figure 7, Table 4 and in Appendix A Table A3 for the commercial device, the discrete and the continuous methods. The Spearman correlation coefficients were high (r > 0.89, *p* < 0.05) for both algorithms and was highest for the discrete method (r > 0.99) compared to the continuous method (r > 0.89). NparLD analysis showed two significant main effects for algorithm (*p* = 3.67 × 10^−15^) and intensity (*p* = 1.09 × 10^−10^), as well as an interaction effect for algorithm × intensity of PA (*p* = 1.32 × 10^−3^). The relative effects (shown on Figure 7B) and post-hoc results show that the differences observed were principally for the continuous method at high levels of PA (Vigorous and Very vigorous). The continuous method differs slightly from the commercially available classification method but allows us to quantify the level of activity more precisely (quantification calculated sample-by-sample vs. second-by-second).

## 4. Discussion

This study presents two different versions of an open-source algorithm (https://github.com/ingreadaptulaval/activitycounts) to compute activity counts (i.e., fixed and modifiable bandwidth algorithms) and two different versions of an activity classification algorithm (i.e., discrete and continuous) and compares them to a popular, commercially available, closed-source activity count algorithm (Actilife^TM^). The fixed and modifiable bandwidth algorithms have been shown to be valid when compared to commercial algorithms (all r > 0.96, *p* < 0.0001) for quantifying activity during different tasks (e.g., activities of daily living, wheelchair propulsion), for a variety of recording durations (i.e., several minutes to several days), in non-disabled participants and manual wheelchair users. The fixed algorithm results are closer to those of the commercial solution than those of the modifiable algorithm (average relative difference of 1.4% and 2.5%, respectively). However, the modifiable algorithm still shows an excellent performance and presents the advantage of being adaptable to pathological populations (e.g., modifying the filter characteristics to minimize the effects of tremors or spasms). In addition, the number of data points out of the limits of agreement was higher for shorter recording durations (project 2: 5%) compared to longer ones (projects 1 and 4: 3.9%; project 3: 2%). This could be explained by the smaller amount of data for project 2, especially when an epoch of 60 s is selected, giving more weight to each error (i.e., one data point outside for 60 s in project 2 represents 10% of the total dataset, while it only represents 0.01% in project 1). The algorithm proposed in the pioneering work of BrØnd & al. [14] showed a similar behavior: these authors reported an average relative difference of 2.2% ± 1.7% and a Cohen’s kappa of 0.945 (meaning an almost perfect level of agreement as stated by [27], but using a more complex algorithm that allowed less adaptability capacity than both algorithms presented in the current study. Indeed, our proposed fixed bandwidth algorithm is simpler (8th- vs. 20th-order filter) than that of BrØnd & al. [10], thereby allowing adaptability to various data sampling rates, and the modifiable bandwidth version allows us to modify the frequency bandwidth to best match the movement profile of clinical populations, environments and tasks.

Adding to these results, the two proposed activity classification methods also showed excellent performance. Indeed, the discrete algorithm reproduced the commercial activity classifier results in an accurate manner, while the continuous method differed from the commercial solution but still reported high concurrent validity (r > 0.89, *p* < 0.05). In fact, the continuous method should be considered more precise to classify levels of physical activity as it operated sample-by-sample (e.g., 100 computations at each second for a sampling rate of 100 Hz) as opposed to the discrete method, which calculates values second-by-second. As they are both valid methods to classify levels of activity, we suggest that the discrete method should be used when comparing results with other studies using commercial solutions, and the continuous method should be used otherwise.

### 4.1. Clinical Implications

Wearable sensors, including accelerometers, are already used with various clinical populations to measure PA in the rehabilitation setting [28]. However, as is generally the case, commercial activity count algorithms are not publicly available (or open-source) as they are protected by trade secret. The development of an open-source activity intensity count algorithm is important to ensure sustainability of data processing as commercially available devices change over time, and to enable researchers to adapt the algorithm to the clinical population under study. Rehabilitation programs and clinical research will benefit from the proposed open-source algorithms as they are more adaptable to the real-world context and therefore become more precise than commercially available solutions when used to assess physical activity in the presence of movement disorders (e.g., spasticity, tremors, slowness of movement).

The proposed algorithms have been validated with different tasks and on different data collection duration time scales, from minutes to weeks. The modifiable bandwidth algorithm can be used with people living with incapacities. For example, in persons with Parkinson’s disease, the band-pass frequency can be adjusted to remove tremors from the signals. For persons having slowness of movements (e.g., stroke survivors), the threshold can be reduced, and on the contrary, in persons living with spasms or hyperkinesia (e.g., cerebral palsy), the threshold can be increased. The continuous method to classify levels of PA is also an interesting tool for clinicians and rehabilitation researchers as it helps to identify the level of PA performed over a given amount of time more precisely. Indeed, people living with disabilities often have to deal with a greater amount of fatigue, which means small periods of time with high levels of intensity. This could lead the commercial algorithm to underestimate their overall physical activity level.

### 4.2. Limitations

First, the proposed algorithms have been tested mainly on healthy subjects; only one population living with a disability (i.e., manual wheelchair users) was evaluated. It will be interesting to test the validity of the different methods proposed on other populations such as stroke survivors and people living with Parkinson’s disease or cerebral palsy, to see the effects of spasticity, tremors or slowness of movements on PA level assessment. Second, it would have been interesting to evaluate the validity of the four proposed algorithms with different brands of accelerometers as in [14] (i.e., they used two different brands: ActiGraph and Axivity). Indeed, in this paper, the proposed algorithm activity counts using the Actigraph raw data were compared to the Actigraph Actilife software activity count. This decision was to make sure that the difference between activity counts came from the algorithm design and not from the fact that two or more sensors would not be exactly located at the same location on the arm or that they could move relative to another. As mentioned in [14], the algorithm can be used with other accelerometer sensors or inertial measurement units (IMU) (which comprise accelerometers), as they are based on raw data acceleration, which is typically very similar between devices. Two main accelerometer characteristics that could lead to differences between raw sensor data are the sensor’s precision (minimum increment between two values, for instance, 0.001 g) and the accelerometer range (for instance, ±8 g). The precision should not be a major issue since the deadband and the bandpass filter in the algorithm will tend to minimize this difference. The range (ex. ±8 g for Actigraph GT9x used in this study) is more problematic. Indeed, if the range is different between sensors (ex. ±2 g vs. ±8 g), the activity count results could differ since one sensor would saturate. The effect of this should be limited since an accelerometer sensor range is typically minimal at ±2 g and the proposed algorithm saturates the signal either way at 2.13 g. In order to minimize discrepancies with the proposed algorithm, one should aim to set the range to ±8 g. Third, as accelerometers detect linear accelerations, they may detect false levels of activity by considering as valid any “false positive” activity counts such as acceleration when driving a car or going up in an elevator. This should be further investigated. Fourth, as accelerometer signals are highly dependent on the limb on which the accelerometer is placed, clinicians will have to carefully choose the placement of their sensors depending on the type of physical activity mostly performed by their patients (e.g., putting sensors on the leg if the patient mostly does cycling). Otherwise, activities might not be considered in the level of physical activity, which could underestimate the extent of physical activity in some patients. Finally, the open-source algorithms made available with this study (https://github.com/ingreadaptulaval/activitycounts) are adapted for research purposes (available in MATLAB format with a user interface that requires a certain level of knowledge in computer science), but are not yet adapted for use in clinical practice. Indeed, as clinicians must perform their assessment in a limited amount of time, they may need an easier-to-use interface, which would require further development.

## 5. Conclusions

This study presents the design and validation of open-source algorithms for activity intensity quantification and classification. They are valid and can be used to assess the PA of manual wheelchair users and healthy participants at different joints (wrist, leg and trunk), during various recording durations (several minutes to days) and for a variety of physical activities. The development of an open-source activity intensity count algorithm is important to ensure sustainability of data processing as wearable devices evolve, and to enable researchers to adapt the data processing algorithm to the needs of various clinical populations. Two versions of the activity intensity quantification are proposed: fixed and modifiable bandwidths. Two versions of the classification algorithm are also proposed: discrete and continuous. The combination of the fixed bandwidth with the discrete method results in a solution close to what is commercially available. On the other hand, the modifiable bandwidth version remains valid while being more adaptable, and the continuous classifying method generates more precise results. Future work will consist of adapting the algorithms to various clinical populations.

## Figures and Tables

**Table 1:** Freedson 3 VM3 count cutpoints [24].

Activity Intensity	Vm3 Counts per Min
Light	<2960
Moderate	2960–6166
Vigorous	6167–9642
Very vigorous	>9642

**Table 2:** Synthesis of data collection characteristics and comparison parameters between the fixed bandwidth algorithm and the commercial solution. “Min.” stands for minute; “dom” stands for dominant; “non-dom” stands for “non-dominant”.

Participant ID	Project	Subject	Length (Min.)	Total Activity Counts	Average Counts per Minute with ActiLife (Counts/Min.)	Average Counts per Minute with Fixed Bandwidth Algo (Counts/Min.)	Absolute Difference (Counts/Min.)	Relative Difference (%)	Correlation (r)	*p*-Value
1	Activities of daily living—24-h data collection	S1	1440.0	5,393,214	3745.3	3672.3	−73.0	−1.95	0.995	<0.001
2		S2	1440.0	4,429,576	3076.1	3027.0	−49.1	−1.60	0.996	<0.001
3		S3	1440.0	1,731,214	1202.2	1176.0	−26.3	−2.18	0.994	<0.001
4		S4	1440.0	4,852,562	3369.8	3294.7	−75.2	−2.23	0.998	<0.001
		Mean (SD)	1440.0 (0.0)	4,101,641 (1,628,753)	2848.4 (1131.1)	2792.5 (1109.7)	−55.9 (23.0)	−2.0 (0.3)	0.996 (0.002)	
5	Bilateral manipulation in daily living tasks	S1 (dom)	12.7	77,511	6095.2	5992.2	−103.0	−1.69	0.994	<0.001
6		S1 (non-dom)	11.3	67,930	6029.3	5888.0	−141.3	−2.34	0.993	<0.001
7		S 2 (dom)	12.0	57,727	4810.6	4732.0	−78.5	−1.63	0.996	<0.001
8		S 2 (non-dom)	11.5	67,304	5869.6	5742.0	−127.6	−2.17	0.995	<0.001
9		S3 (dom)	67.0	176,932	2640.8	2591.4	−49.4	−1.87	0.995	<0.001
10		S3 (non-dom)	67.0	186,388	2781.9	2743.7	−38.2	−1.37	0.995	<0.001
11		S4 (dom)	74.7	148,068	1981.3	1965.1	−16.1	−0.81	0.996	<0.001
12		S4 (non-dom)	74.3	161,167	2169.6	2140.7	−28.9	−1.33	0.996	<0.001
13		S5 (dom)	63.5	234,319	3691.0	3612.2	−78.8	−2.14	0.995	<0.001
14		S5 (non-dom)	62.8	239,083	3805.0	3728.3	−76.7	−2.02	0.996	<0.001
15		S6 (dom)	10.3	84,440	8184.8	7912.1	−272.7	−3.33	0.994	<0.001
16		S6 (non-dom)	9.9	73,246	7423.6	7233.8	−189.8	−2.56	0.996	<0.001
17		S7 (dom)	11.0	68,910	6264.6	6114.8	−149.8	−2.39	0.995	<0.001
18		S7 (non-dom)	10.5	62,272	5921.3	5782.9	−138.4	−2.34	0.993	<0.001
19		S8 (dom)	14.1	106,057	7548.6	7333.3	−215.2	−2.85	0.994	<0.001
20		S8 (non-dom)	14.6	102,027	7012.2	6843.6	−168.6	−2.40	0.995	<0.001
21		S9 (dom)	63.0	152,663	2423.2	2415.1	−8.1	−0.33	0.996	<0.001
22		S9 (non-dom)	63.0	142,499	2261.9	2249.4	−12.5	−0.55	0.996	<0.001
23		S10 (dom)	64.6	178,064	2758.5	2714.7	−43.9	−1.59	0.995	<0.001
24		S10 (non-dom)	65.3	145,858	2234.2	2206.4	−27.8	−1.25	0.995	<0.001
25		S11 (dom)	64.1	217,897	3399.3	3318.7	−80.6	−2.37	0.996	<0.001
26		S11 (non-dom)	63.5	242,062	3812.0	3733.6	−78.4	−2.06	0.997	<0.001
		Mean (SD)	41.4 (27.9)	136,019 (62,835)	4505.4 (2049.5)	4408.8 (1980.9)	−96.6 (71.5)	−1.9 (0.47)	0.995 (0.001)	
27	Manual wheelchair propulsion	S1 (day 1)	18,751.2	7,343,724	391.6	393.5	1.9	0.49	0.992	<0.001
28		S1 (day 2)	41,371.6	5,119,126	123.7	124.2	0.4	0.36	0.993	<0.001
29		S2 (day 1)	17,408.7	4,030,397	231.5	231.7	0.2	0.10	0.994	<0.001
30		S2 (day 2)	Missing data							
31		S3 (day 1)	12,712.8	4,587,229	360.8	361.6	0.8	0.21	0.993	<0.001
32		S3 (day 2)	17,618.3	7,359,802	417.7	416.8	−0.9	−0.22	0.993	<0.001
33		S4 (day 1)	15,836.5	7,818,844	493.7	496.4	2.7	0.55	0.994	<0.001
34		S4 (day 2)	40,495.5	11,001,481	271.7	273.1	1.5	0.54	0.994	<0.001
35		S5 (day 1)	13,756.9	6,218,452	452.0	453.3	1.3	0.29	0.992	<0.001
36		S5 (day 2)	22,635.8	9,809,569	433.4	433.6	0.2	0.05	0.993	<0.001
37		S6 (day 1)	34,020.7	3,070,352	90.2	90.1	−0.1	−0.15	0.992	<0.001
38		S6 (day 2)	17,185.5	3,340,505	194.4	194.6	0.2	0.10	0.992	<0.001
39		S7 (day 1)	17,280.0	6,578,440	380.7	381.3	0.6	0.15	0.991	<0.001
40		S7 (day 2)	9916.3	3,359,621	338.8	339.2	0.4	0.12	0.991	<0.001
		Mean (SD)	21,460.8 (10,380.1)	6,125,964 (2,515,702.0)	321.6 (128.5)	322.3 (128.9)	0.7 (0.9)	0.2 (0.2)	0.993 (0.001)	
41	Real-world working environment—Nordic expedition project on the Amundsen	S1 (leg)	32,025.6	2,128,9876	664.8	678.8	14.0	2.10	0.995	<0.001
42		S1 (trunk)	33,529.6	17,771,319	530.0	543.0	12.9	2.44	0.995	<0.001
43		S2 (leg)	34,883.1	20,414,335	585.2	595.8	10.6	1.81	0.995	<0.001
44		S2 (trunk)	30,109.1	16,771,642	557.0	567.8	10.8	1.93	0.995	<0.001
		Mean (SD)	32,636.8 (2049.9)	19,061,792 (2,137,275.3)	584.3 (58.2)	596.3 (59.0)	12.1 (1.7)	2.1 (0.3)	0.996 (0.0018)	

**Table 3:** Summary of data collection characteristics and comparison parameters between the modifiable bandwidth algorithm and the commercial solution.

Participant ID	Project	Subject	Length (Min.)	Total Activity Counts	Average Counts per Minute with Actilife (Counts/Min.)	Average Counts per Minute with Modifiable bandwith algo (Counts/Min.)	Absolute Difference (Counts/Min.)	Relative Difference (%)	Correlation (r)	*p*-Value
1	Activities of daily living—24-h data collection	S1	1440.0	5,393,214	3745.3	3842.3	97.1	2.59	0.987	<0.001
2		S2	1440.0	4,429,576	3076.1	3130.6	54.5	1.77	0.989	<0.001
3		S3	1440.0	1,731,214	1202.2	1229.9	27.7	2.30	0.981	<0.001
4		S4	1440.0	4,852,562	3369.8	3392.2	22.4	0.67	0.995	<0.001
		Mean (SD)	1440.0 (0.0)	4,101,641 (1,628,753)	2848.4 (1131.1)	2898.8 (1150.7)	50.4 (34.1)	1.8 (0.8)	0.988 (0.006)	
5	Bilateral manipulation in daily living tasks	S1 (dom)	12.7	77,511	6095.2	6380.2	285.0	4.68	0.983	<0.001
6		S1 (non-dom)	11.3	67,930	6029.3	6324.5	295.2	4.90	0.982	<0.001
7		S2 (dom)	12.0	57,727	4810.6	4890.6	80.0	1.66	0.986	<0.001
8		S2 (non-dom)	11.5	67,304	5869.6	6048.7	179.2	3.05	0.983	<0.001
9		S3 (dom)	67.0	176,932	2640.8	2693.3	52.5	1.99	0.986	<0.001
10		S3 (non-dom)	67.0	186,388	2781.9	2876.1	94.2	3.39	0.988	<0.001
11		S4 (dom)	74.7	148,068	1981.3	2045.0	63.7	3.21	0.990	<0.001
12		S4 (non-dom)	74.3	161,167	2169.6	2226.7	57.1	2.63	0.991	<0.001
13		S5 (dom)	63.5	234,319	3691.0	3743.2	52.2	1.41	0.985	<0.001
14		S5 (non-dom)	62.8	239,083	3805.0	3917.3	112.3	2.95	0.987	<0.001
15		S6 (dom)	10.3	84,440	8184.8	8271.7	86.9	1.06	0.989	<0.001
16		S6 (non-dom)	9.9	73,246	7423.6	7537.9	114.3	1.54	0.990	<0.001
17		S7 (dom)	11.0	68,910	6264.6	6391.0	126.4	2.02	0.982	<0.001
18		S7 (non-dom)	10.5	62,272	5921.3	6094.8	173.5	2.93	0.979	<0.001
19		S8 (dom)	14.1	106,057	7548.6	7906.1	357.5	4.74	0.986	<0.001
20		S8 (non-dom)	14.6	102,027	7012.2	7370.7	358.5	5.11	0.989	<0.001
21		S9 (dom)	63.0	152,663	2423.2	2548.5	125.3	5.17	0.990	<0.001
22		S9 (non-dom)	63.0	142,499	2261.9	2368.2	106.4	4.70	0.990	<0.001
23		S10 (dom)	64.6	178,064	2758.5	2766.6	8.1	0.29	0.987	<0.001
24		S10 (non-dom)	65.3	145,858	2234.2	2242.0	7.7	0.35	0.988	<0.001
25		S11 (dom)	64.1	217,897	3399.3	3400.8	1.5	0.04	0.993	<0.001
26		S11 (non-dom)	63.5	242,062	3812.0	3857.9	45.9	1.20	0.994	<0.001
		Mean (SD)	41.4 (27.9)	136,019 (62,835)	4505.4 (2049.5)	4631.9 (2121.7)	126.5 (107.2)	2.7 (1.7)	0.987 (0.004)	
27	Manual wheelchair propulsion	S1 (day 1)	18,751.2	7,343,724	391.6	389.3	−2.3	−0.59	0.966	<0.001
28		S1 (day 2)	41,371.6	5,119,126	123.7	123.9	0.2	0.13	0.972	<0.001
29		S2 (day 1)	17,408.7	4,030,397	231.5	241.9	10.4	4.48	0.983	<0.001
30		S2 (day 2)	Missing data							
31		S3 (day 1)	12,712.8	4,587,229	360.8	366.3	5.5	1.52	0.972	<0.001
32		S3 (day 2)	17,618.3	7,359,802	417.7	424.7	6.9	1.66	0.972	<0.001
33		S4 (day 1)	15,836.5	7,818,844	493.7	506.3	12.6	2.55	0.979	<0.001
34		S4 (day 2)	40,495.5	11,001,481	271.7	278.7	7.1	2.60	0.980	<0.001
35		S5 (day 1)	13,756.9	6,218,452	452.0	448.3	−3.7	−0.83	0.965	<0.001
36		S5 (day 2)	22,635.8	9,809,569	433.4	432.5	−0.9	−0.21	0.969	<0.001
37		S6 (day 1)	34,020.7	3,070,352	90.2	88.2	−2.1	−2.27	0.965	<0.001
38		S6 (day 2)	17,185.5	3,340,505	194.4	189.3	−5.1	−2.61	0.964	<0.001
39		S7 (day 1)	17,280.0	6,578,440	380.7	374.9	−5.8	−1.51	0.960	<0.001
40		S7 (day 2)	9916.3	3,359,621	338.8	336.1	−2.7	−0.81	0.965	<0.001
		Mean (SD)	21,460.8 (10,380.1)	6,125,964 (2,515,701)	321.6 (128.5)	323.1 (129.7	1.5 (6.2)	0.3 (2.1)	0.97 (0.007)	
41	Real-world working environment—Nordic expedition project on the Amundsen	S1 (leg)	32,025.6	2,128,9876	664.8	693.4	28.6	4.31	0.988	<0.001
42		S1 (trunk)	33,529.6	17,771,319	530.0	543.9	13.9	2.62	0.981	<0.001
43		S2 (leg)	34,883.1	20,414,335	585.2	615.2	30.0	5.12	0.988	<0.001
44		S2 (trunk)	30,109.1	16,771,642	557.0	580.8	23.8	4.27	0.982	<0.001
		Mean (SD)	32,636.8 (2049.9)	19,061,792 (2,137,275)	584.3 (58.2)	608.3 (63.8)	24.1 (7.3)	4.1 (1.0)	0.985 (0.004)	

**Table 4:** Post-hoc results for the activity intensity classification compared to the classification of the commercial solution (*p*-value, statistically significant results are in bold).

Algorithm	Level of Physical Activity			
	Light	Moderate	Vigorous	Very vigorous
**Discrete**	0.032	0.254	0.010	0.052
**Continuous**	0.580	0.249	1.78 × 10^−5^	9.9 × 10^−8^

Legend: Statically significant results are identified in bold (*p* < 0.00625 with Bonferroni corrections).

## Appendix A

**Table A1:** A synthesis of the activities performed by subjects of the 24-h project (project 1).

*Activities*	Number of Reporting Participants/4
*Desk work*	4
*Leisure time*	4
*Spikeball*	1
*Walking*	3
*Activities of daily living (cooking, cleaning up, etc.)*	4
*Playing guitar*	1
*Playing with the dog*	2
*Driving*	4
*Getting dressed*	4
*Sleeping*	4
*Watching television*	2
*Brushing teeth*	4
*Taking the bus*	2
*Running*	1

**Table A2:** Number of data points outside of the limits of agreement, regression coefficient, mean difference, upper and lower limits of agreement for the fixed and the modifiable algorithm for epochs of 1 s, 10 s, 60 s. “s” stands for seconds.

Participant-ID	Project	Participant	Epoch	*n* Total	*n* Outside—Fixed (Absolute Value)	*n* Outside—Modifiable (Absolute Value)	*n* Outside—Fixed (%)	*n* Outside-Modifiable (%)	Regression Coefficient—Fixed	Regression Coefficient—Modifiable	Mean of Difference (Bias) —Fixed	LoA—Fixed	UpLoA—Fixed	DownLoA—Fixed	Mean of Difference (Bias)-Modifiable	LoA-Modifiable	UpLoA-Modifiable	DownLoA-Modifiable
1	Activities of daily living—24-h data collection	Subject 1	1 s	86 400	2252	2800	2.6	3.2	0.66	0.58	−1.22	29.69	28.48	−30.91	1.62	48.55	50.16	−46.93
			10 s	8640	300	316	3.5	3.7	0.73	0.77	−11.30	76.87	65.57	−88.17	16.23	112.80	129.03	−96.57
			60 s	1440	61	66	4.2	4.6	0.84	0.81	−63.99	267.91	203.92	−331.90	94.77	372.19	466.96	−277.41
2		Subject 2	1 s	86 400	2117	2286	2.5	2.6	0.66	0.51	−0.82	25.14	24.32	−25.96	0.91	43.03	43.94	−42.12
			10 s	8640	258	293	3.0	3.4	0.73	0.61	−7.65	67.33	59.68	−74.98	9.52	97.93	107.46	−88.41
			60 s	1440	42	62	2.9	4.3	0.84	0.64	−43.42	233.69	190.27	−277.11	55.18	278.64	333.82	−223.47
3		Subject 3	1 s	86,400	1424	1549	1.6	1.8	0.63	0.51	−0.44	22.39	21.95	−22.83	0.46	39.90	40.36	−39.44
			10 s	8640	252	279	2.9	3.2	0.69	0.61	−4.17	48.75	44.58	−52.93	4.61	78.65	83.26	−74.03
			60 s	1440	57	65	4.0	4.5	0.75	0.64	−23.79	118.17	94.38	−141.96	26.28	188.43	214.70	−162.15
4		Subject 4	1 s	86,400	1753	1890	2.0	2.2	0.63	0.42	−1.25	23.76	22.51	−25.02	0.37	39.25	39.63	−38.88
			10 s	8640	259	232	3.0	2.7	0.69	0.41	−12.13	83.24	71.11	−95.37	3.95	88.78	92.72	−84.83
			60 s	1440	66	46	4.6	3.2	0.75	0.41	−71.14	369.01	297.87	−440.15	22.03	232.05	254.08	−210.02
		Mean (SD)	1 s		1886.5 (373.5)	2131.3 (538.0)	2.2 (0.4)	2.5 (0.6)	0.6 (0.02)	0.5 (0.07)	−0.9 (0.4)	25.2 (3.2)	24.3 (3.0)	−26.2 (3.4)	0.8 (0.6)	42.7 (4.2)	43.5 (4.8)	−41.8 (3.7)
			10 s		367.3 (22.1)	280.0 (35.4)	3.1 (0.3)	3.2 (0.4)	0.7 (0.02)	0.6 (0.15)	−8.8 (3.7)	69.0 (15.0)	60.2 (11.4)	−77.9 (18.6)	8.6 (5.7)	94.5 (14.5)	103.1 (19.9)	−86.0 (9.3)
			60 s		56.5 (10.3)	59.8 (9.3)	3.9 (0.7)	4.1 (0.6)	0.8 (0.05)	0.6 (0.16)	−50.6 (21.4)	247.2 (103.4)	196.6 (83.3)	−297.8 (124.0)	49.6 (33.5)	267.8 (78.7)	317.4 (111.3)	−218.3 (47.4)
5	Bilateral manipulation in daily living tasks	Subject 1 (dom)	1 s	763	39	39	5.1	5.1	0.49	0.37	−1.72	27.47	25.75	−29.18	4.75	46.46	51.21	−41.71
			10 s	76	4	6	5.3	7.9	0.65	0.78	−17.30	68.37	51.06	−85.67	50.74	130.50	181.25	−79.76
			60 s	12	0	0	0.0	0.0	0.69	0.76	−102.82	157.86	55.04	−260.68	303.62	397.02	700.64	−93.39
6		Subject 1 (non-dom)	1 s	676	41	30	6.1	4.4	0.50	0.32	−2.36	28.32	25.97	−30.68	4.92	48.15	53.07	−43.23
			10 s	67	4	3	6.0	4.5	0.53	0.74	−22.86	67.89	45.03	−90.75	52.68	170.42	223.10	−117.73
			60 s	11	0	0	0.0	0.0	0.67	0.48	−128.84	156.63	27.79	−285.48	320.92	320.34	641.26	0.57
7		Subject 2 (dom)	1 s	720	21	22	2.9	3.1	0.56	0.46	−1.31	27.56	26.26	−28.87	1.33	49.25	50.58	−47.91
			10 s	72	4	3	5.6	4.2	0.58	0.56	−13.16	53.20	40.04	−66.35	13.84	95.86	109.70	−82.02
			60 s	12	1	0	8.3	0.0	0.47	0.76	−78.28	127.29	49.01	−205.57	78.85	236.89	315.74	−158.05
8		Subject 2 (non-dom)	1 s	688	26	23	3.8	3.3	0.50	0.41	−2.13	31.12	29.00	−33.25	2.99	56.99	59.98	−54.01
			10 s	68	2	3	2.9	4.4	0.55	0.46	−20.30	73.02	52.72	−93.32	32.29	100.94	133.23	−68.65
			60 s	11	0	0	0.0	0.0	0.65	0.74	−114.73	181.98	67.25	−296.71	185.52	196.68	382.20	−11.17
9		Subject 3 (dom)	1 s	4020	131	132	3.3	3.3	0.66	0.57	−0.82	24.79	23.97	−25.61	0.88	41.18	42.05	−40.30
			10 s	402	23	21	5.7	5.2	0.73	0.69	−7.96	60.37	52.41	−68.32	8.80	83.57	92.38	−74.77
			60 s	67	5	3	7.5	4.5	0.69	0.83	−45.64	182.24	136.60	−227.88	52.02	251.52	303.54	−199.49
10		Subject 3 (non-dom)	1 s	4020	118	120	2.9	3.0	0.69	0.55	−0.64	25.01	24.38	−25.65	1.57	39.65	41.22	−38.08
			10 s	402	25	21	6.2	5.2	0.76	0.73	−6.51	56.11	49.60	−62.62	15.86	89.14	105.00	−73.29
			60 s	67	6	5	9.0	7.5	0.75	0.84	−34.77	146.30	111.53	−181.07	91.77	272.18	363.95	−180.40
11		Subject 4 (dom)	1 s	4484	100	100	2.2	2.2	0.65	0.57	−0.27	22.59	22.32	−22.86	1.06	33.28	34.34	−32.22
			10 s	448	18	16	4.0	3.6	0.72	0.69	−2.77	51.08	48.31	−53.86	10.21	69.29	79.51	−59.08
			60 s	74	3	2	4.1	2.7	0.72	0.71	−16.55	135.99	119.44	−152.54	56.91	189.30	246.21	−132.40
12		Subject 4 (non-dom)	1 s	4457	103	114	2.3	2.6	0.65	0.53	−0.48	24.33	23.85	−24.82	0.95	34.22	35.17	−33.27
			10 s	445	15	21	3.4	4.7	0.72	0.70	−4.90	59.42	54.53	−64.32	8.98	76.27	85.25	−67.28
			60 s	74	3	3	4.1	4.1	0.72	0.72	−27.42	140.47	113.05	−167.89	52.51	235.90	288.42	−183.39
13		Subject 5 (dom)	1 s	3809	122	160	3.2	4.2	0.63	0.53	−1.31	26.09	24.78	−27.41	0.87	45.47	46.34	−44.60
			10 s	380	19	31	5.0	8.2	0.74	0.70	−12.53	76.88	64.34	−89.41	9.75	142.70	152.45	−132.94
			60 s	63	3	2	4.8	3.2	0.83	0.72	−74.78	267.77	192.99	−342.56	51.58	500.24	551.82	−448.66
14		Subject 5 (non-dom)	1 s	3770	143	146	3.8	3.9	0.63	0.56	−1.28	25.53	24.25	−26.80	1.87	44.93	46.80	−43.06
			10 s	377	20	25	5.3	6.6	0.74	0.77	−11.87	71.00	59.13	−82.86	19.89	145.03	164.92	−125.15
			60 s	62	3	2	4.8	3.2	0.83	0.79	−67.94	235.51	167.58	−303.45	110.93	561.66	672.59	−450.73
15		Subject 6 (dom)	1 s	619	25	29	4.0	4.7	0.49	0.56	−4.55	37.87	33.32	−42.41	1.45	50.73	52.18	−49.28
			10 s	61	3	5	4.9	8.2	0.49	0.77	−42.65	89.98	47.33	−132.63	15.59	106.10	121.69	−90.51
			60 s	10	0	0	0.0	0.0	0.57	0.79	−251.43	270.71	19.29	−522.14	98.72	288.69	387.41	−189.96
16		Subject 6 (non-dom)	1 s	592	35	30	5.9	5.1	0.49	0.44	−3.16	33.22	30.06	−36.38	1.90	49.08	50.99	−47.18
			10 s	59	2	6	3.4	10.2	0.49	0.31	−27.20	76.06	48.87	−103.26	23.89	97.50	121.38	−73.61
			60 s	9	1	0	11.1	0.0	0.57	0.46	−152.90	132.53	−20.37	−285.43	152.08	216.44	368.52	−64.36
17		Subject 7 (dom)	1 s	660	25	32	3.8	4.8	0.47	0.44	−2.50	26.14	23.65	−28.64	2.11	52.05	54.16	−49.95
			10 s	66	1	3	1.5	4.5	0.68	0.31	−23.83	75.22	51.39	−99.05	25.49	117.64	143.14	−92.15
			60 s	11	1	0	9.1	0.0	0.81	0.46	−124.88	197.00	72.12	−321.88	144.73	210.82	355.56	−66.09
18		Subject 7 (non-dom)	1 s	631	27	31	4.3	4.9	0.47	0.36	−2.31	28.05	25.75	−30.36	2.89	52.26	55.15	−49.37
			10 s	63	4	5	6.3	7.9	0.68	0.64	−23.15	71.47	48.31	−94.62	33.19	109.59	142.78	−76.39
			60 s	10	0	0	0.0	0.0	0.81	0.90	−121.93	211.99	90.06	−333.91	199.77	209.77	409.54	−10.00
19		Subject 8 (dom)	1 s	843	37	48	4.4	5.7	0.55	0.36	−3.59	34.99	31.40	−38.58	5.96	54.97	60.92	−49.01
			10 s	84	4	5	4.8	6.0	0.55	0.64	−34.13	79.72	45.60	−113.85	60.91	160.28	221.20	−99.37
			60 s	14	0	0	0.0	0.0	0.56	0.90	−200.15	157.23	−42.92	−357.38	339.18	322.57	661.76	16.61
20		Subject 8 (non-dom)	1 s	873	41	48	4.7	5.5	0.55	0.47	−2.81	35.64	32.83	−38.45	5.97	54.13	60.11	−48.16
			10 s	87	3	4	3.4	4.6	0.55	0.71	−27.53	79.79	52.26	−107.33	60.51	165.10	225.60	−104.59
			60 s	14	1	0	7.1	0.0	0.56	0.75	−153.51	167.61	14.09	−321.12	359.27	340.18	699.45	19.08
21		Subject 9 (dom)	1 s	3780	105	115	2.8	3.0	0.63	0.47	−0.14	19.28	19.14	−19.41	2.09	29.70	31.78	−27.61
			10 s	378	17	20	4.5	5.3	0.68	0.71	−1.99	51.84	49.84	−53.83	21.09	81.03	102.12	−59.94
			60 s	63	6	3	9.5	4.8	0.73	0.75	−9.26	139.64	130.38	−148.89	120.88	323.49	444.38	−202.61
22		Subject 9 (non-dom)	1 s	3 780	116	120	3.1	3.2	0.63	0.61	−0.21	18.53	18.32	−18.74	1.77	30.01	31.78	−28.24
			10 s	378	18	17	4.8	4.5	0.68	0.83	−2.14	48.71	46.57	−50.86	17.65	79.68	97.33	−62.02
			60 s	63	4	4	6.3	6.3	0.73	0.86	−11.02	150.35	139.34	−161.37	102.11	291.27	393.38	−189.15
23		Subject 10 (dom)	1 s	3 873	127	123	3.3	3.2	0.65	0.57	−0.73	21.33	20.60	−22.06	0.13	34.83	34.96	−34.69
			10 s	387	20	21	5.2	5.4	0.68	0.74	−6.24	59.72	53.48	−65.96	2.11	106.98	109.08	−104.87
			60 s	64	3	3	4.7	4.7	0.57	0.84	−33.45	190.63	157.18	−224.07	12.36	450.05	462.41	−437.69
24		Subject 10 (non-dom)	1 s	3917	118	130	3.0	3.3	0.65	0.61	−0.46	20.84	20.38	−21.30	0.13	31.37	31.50	−31.24
			10 s	391	12	17	3.1	4.3	0.68	0.83	−3.66	57.06	53.40	−60.71	2.26	82.74	85.01	−80.48
			60 s	65	5	4	7.7	6.2	0.57	0.86	−19.58	180.17	160.59	−199.75	15.23	271.48	286.71	−256.26
25		Subject 11 (dom)	1 s	3846	100	135	2.6	3.5	0.69	0.57	−1.34	29.53	28.19	−30.88	0.02	38.67	38.69	−38.64
			10 s	384	21	21	5.5	5.5	0.81	0.74	−12.73	80.82	68.09	−93.55	0.20	113.04	113.24	−112.84
			60 s	64	4	4	6.3	6.3	0.85	0.84	−72.27	285.53	213.27	−357.80	1.35	471.13	472.48	−469.78
26		Subject 11 (non-dom)	1 s	3 810	105	150	2.8	3.9	0.69	0.60	−1.31	29.62	28.32	−30.93	0.76	38.88	39.64	−38.11
			10 s	381	20	25	5.2	6.6	0.81	0.61	−11.09	73.59	62.50	−84.69	9.23	104.37	113.60	−95.14
			60 s	63	4	4	6.3	6.3	0.85	0.84	−61.68	254.06	192.38	−315.75	53.71	420.49	474.21	−366.78
		Mean (SD)	1 s		77.5 (44.2)	85.3 (50.6)	3.6 (1.1)	3.9 (1.0)	0.6 (0.08)	0.5 (0.09)	−1.6 (0.5)	27.2 (4.3)	25.6(3.9)	−28.8 (4.8)	2.1 (0.4)	43.5 (4.9)	45.6 (5.3)	−41.4 (4.5)
			10 s		11.8 (8.5)	13.6 (9.2)	4.6 (1.2)	5.8 (1.7)	0.7 (0.1)	0.7 (0.1)	−15.3 (5.2)	67.3 (23.5)	52.0 (18.3)	−82.6 (28.6)	22.5 (4.0)	110.4 (16.9)	132.9 (20.7)	−87.8 (13.3)
			60 s		2.4 (2.1)	1.8 (1.8)	5.0 (3.6)	2.7 (2.8)	0.7 (0.1)	0.8 (0.1)	−86.5 (30.4)	185.0 (137.2)	98.4 (107.3)	−271.5 (167.3)	132.0 (23.6)	317.2 (63.7)	449.2 (86.1)	−185.2 (42.6)
27	Manual wheelchair propulsion	Subject 1 (day 1)	1 s	1,125,071	8490	10,351	0.8	0.9	0.61	0.42	0.03	15.41	15.45	−15.38	−0.04	31.50	31.46	−31.54
			10 s	112,507	1595	1795	1.4	1.6	0.65	0.41	0.08	28.93	29.01	−28.84	−0.33	58.47	58.14	−58.79
			60 s	18,751	499	489	2.7	2.6	0.67	0.41	0.55	57.77	58.32	−57.21	−2.45	129.08	126.63	−131.54
28		Subject 1 (day 2)	1 s	2,482,293	5670	6773	0.2	0.3	0.61	0.65	0.01	15.27	15.27	−15.26	0.00	30.98	30.98	−30.98
			10 s	248,229	1027	1184	0.4	0.5	0.65	0.76	0.03	27.95	27.98	−27.92	0.04	61.89	61.93	−61.85
			60 s	41,371	301	244	0.7	0.6	0.67	0.76	0.21	56.35	56.55	−56.14	0.09	153.76	153.85	−153.66
29		Subject 2 (day 1)	1 s	1,044,523	3828	4608	0.4	0.4	0.64	0.65	0.00	16.47	16.48	−16.47	0.17	28.22	28.39	−28.04
			10 s	104,452	674	1012	0.6	1.0	0.67	0.76	0.00	31.42	31.42	−31.42	1.62	57.90	59.52	−56.27
			60 s	17,408	182	258	1.0	1.5	0.76	0.76	0.15	70.27	70.42	−70.12	9.26	168.46	177.71	−159.20
30		Subject 2 (day 2)	1 s	Missing data														
			10 s															
			60 s															
31		Subject 3 (day 1)	1 s	762,770	5156	6222	0.7	0.8	0.64	0.62	0.01	15.71	15.72	−15.70	0.09	31.16	31.25	−31.07
			10 s	76,277	941	1206	1.2	1.6	0.67	0.76	−0.05	29.60	29.55	−29.65	0.91	57.26	58.17	−56.35
			60 s	12,712	284	285	2.2	2.2	0.76	0.79	−0.26	59.62	59.36	−59.87	5.02	128.20	133.21	−123.18
32		Subject 3 (day 2)	1 s	1,057,088	8287	9709	0.8	0.9	0.62	0.62	−0.02	15.75	15.74	−15.77	0.12	31.78	31.90	−31.67
			10 s	105,709	1506	1815	1.4	1.7	0.64	0.76	−0.23	30.84	30.61	−31.06	1.10	59.60	60.70	−58.50
			60 s	17,618	491	476	2.8	2.7	0.66	0.79	−1.25	63.89	62.64	−65.14	6.10	140.22	146.33	−134.12
33		Subject 4 (day 1)	1 s	950,190	9435	10,331	1.0	1.1	0.62	0.63	0.05	14.33	14.37	−14.28	0.21	26.62	26.83	−26.41
			10 s	95,019	1507	1764	1.6	1.9	0.64	0.76	0.21	26.98	27.19	−26.77	1.94	53.25	55.19	−51.31
			60 s	15,836	360	430	2.3	2.7	0.66	0.80	1.33	58.34	59.67	−57.00	11.00	135.02	146.02	−124.02
34		Subject 4 (day 2)	1 s	2,429,731	12,353	13,603	0.5	0.6	0.66	0.63	0.02	14.64	14.67	−14.62	0.12	27.08	27.20	−26.96
			10 s	242,973	2026	2466	0.8	1.0	0.71	0.76	0.17	27.94	28.10	−27.77	1.09	53.90	54.99	−52.82
			60 s	40,495	573	678	1.4	1.7	0.76	0.80	1.08	61.84	62.92	−60.75	6.22	134.04	140.26	−127.82
35		Subject 5 (day 1)	1 s	825,415	7487	9061	0.9	1.1	0.61	0.65	0.02	15.40	15.42	−15.38	−0.06	31.69	31.62	−31.75
			10 s	82,541	1549	1693	1.9	2.1	0.66	0.76	−0.02	30.03	30.01	−30.06	−0.54	62.58	62.04	−63.12
			60 s	13,756	396	282	2.9	2.1	0.70	0.74	−0.16	67.00	66.84	−67.17	−3.86	161.31	157.45	−165.17
36		Subject 5 (day 2)	1 s	1,358,148	10,654	12,879	0.8	0.9	0.61	0.65	0.00	16.01	16.01	−16.00	−0.01	33.47	33.45	−33.48
			10 s	135,814	2295	2435	1.7	1.8	0.66	0.76	−0.10	30.21	30.11	−30.31	−0.05	62.44	62.39	−62.49
			60 s	22,635	570	507	2.5	2.2	0.68	0.74	−0.50	66.00	65.50	−66.50	−0.71	148.57	147.86	−149.28
37		Subject 6 (day 1)	1 s	2,041,241	4378	4939	0.2	0.2	0.61	0.68	0.00	14.85	14.85	−14.85	−0.03	30.79	30.76	−30.82
			10 s	204,124	784	811	0.4	0.4	0.66	0.76	−0.05	28.02	27.97	−28.08	−0.30	67.74	67.44	−68.04
			60 s	34,020	237	183	0.7	0.5	0.68	0.75	−0.32	55.07	54.74	−55.39	−1.82	170.07	168.24	−171.89
38		Subject 6 (day 2)	1 s	1,031,130	4568	5172	0.4	0.5	0.63	0.68	0.00	15.49	15.49	−15.49	−0.08	31.50	31.41	−31.58
			10 s	103,113	901	1090	0.9	1.1	0.71	0.76	−0.04	29.41	29.36	−29.45	−0.74	67.54	66.81	−68.28
			60 s	17,185	267	263	1.6	1.5	0.76	0.75	−0.24	59.53	59.29	−59.77	−4.38	161.83	157.45	−166.21
39		Subject 7 (day 1)	1 s	1,036,800	7948	10,122	0.8	1.0	0.63	0.65	0.01	15.57	15.57	−15.56	−0.10	31.35	31.25	−31.44
			10 s	103,680	1460	1664	1.4	1.6	0.71	0.75	−0.17	28.91	28.74	−29.08	−0.80	63.28	62.47	−64.08
			60 s	17,280	462	356	2.7	2.1	0.76	0.77	−0.95	54.34	53.39	−55.29	−4.94	158.31	153.37	−163.26
40		Subject 7 (day 2)	1 s	594,979	4340	5238	0.7	0.9	0.61	0.65	0.01	15.07	15.07	−15.06	−0.05	29.49	29.45	−29.54
			10 s	59,497	865	952	1.5	1.6	0.63	0.75	−0.11	27.56	27.45	−27.67	−0.34	63.70	63.36	−64.05
			60 s	9916	230	142	2.3	1.4	0.68	0.77	−0.66	55.45	54.80	−56.11	−2.17	177.80	175.63	−179.98
		Mean (SD)	1 s		7122.6 (2705.7)	8385.2 (3081.5)	0.6 (0.3)	0.7 (0.3)	0.6 (0.02)	0.6 (0.07)	0.0 (0.6)	15.4 (4.5)	15.4 (4.0)	−15.4 (5.0)	0.0 (0.3)	30.4 (4.8)	30.5 (5.1)	−30.4 (4.6)
			10 s		1317.7 (498.6)	1529.8 (538.8)	1.2 (0.5)	1.4 (0.5)	0.7 (0.03)	0.7 (0.1)	0.0 (5.7)	29.1 (25.9)	29.0 (20.1)	−29.1 (31.6)	0.3 (2.6)	60.7 (14.3)	61.0 (16.7)	−60.5 (12.0)
			60 s		373.2 (134.5)	353.3 (153.0)	2.0 (0.8)	1.8 (0.7)	0.7 (0.04)	0.7 (0.1)	−0.1 (33.7)	60.4 (148.6)	60.3(1115.4)	−60.5 (182.0)	1.3 (14.8)	151.3 (44.7)	152.6 (57.8)	−149.9 (33.0)
41	Real-world working environment—Nordic expedition project on the Amundsen	Subject 1 (leg)	1 s	1,921,533	19,170	25,718	1.0	1.3	0.64	0.60	0.23	11.71	11.94	−11.48	0.48	18.28	18.76	−17.80
			10 s	192,153	5037	2016	2.6	1.0	0.77	0.61	2.07	23.25	25.32	−21.18	4.42	66.02	70.44	−61.60
			60 s	32,025	1308	216	4.1	0.7	0.87	0.84	12.04	70.93	82.97	−58.89	25.71	265.87	291.58	−240.15
42		Subject 1 (trunk)	1 s	2,011,773	19,077	25,168	0.9	1.3	0.64	0.66	0.22	10.79	11.00	−10.57	0.23	20.64	20.87	−20.41
			10 s	201,177	4899	1359	2.4	0.7	0.77	0.82	1.83	24.83	26.65	−23.00	1.93	75.18	77.11	−73.25
			60 s	33,529	1212	162	3.6	0.5	0.87	0.81	10.27	76.05	86.32	−65.77	10.31	311.43	321.74	−301.12
43		Subject 2 (leg)	1 s	2,092,986	18,918	23,081	0.9	1.1	0.64	0.58	0.18	11.78	11.96	−11.60	0.50	18.41	18.91	−17.91
			10 s	209,298	4939	5241	2.4	2.5	0.79	0.76	1.60	25.09	26.69	−23.49	4.75	46.42	51.17	−41.67
			60 s	34,883	1357	1333	3.9	3.8	0.89	0.85	9.41	75.58	84.99	−66.17	27.68	150.07	177.75	−122.39
44		Subject 2 (trunk)	1 s	1,806,544	17,820	21,828	1.0	1.2	0.64	0.58	0.18	11.57	11.75	−11.39	0.40	20.53	20.93	−20.13
			10 s	180,654	4888	3839	2.7	2.1	0.79	0.77	1.54	27.23	28.76	−25.69	3.56	50.82	54.38	−47.25
			60 s	30,109	1192	676	4.0	2.2	0.89	0.81	8.61	81.15	89.76	−72.54	19.79	161.09	180.89	−141.30
		Mean (SD)	1 s		18,746.3 (626.2)	23,948.8 (1813.6)	1.0 (0.04)	1.2 (0.1)	0.6 (0.001)	0.6 (0.04)	0.2 (0.6)	11.5 (4.1)	11.7 (3.4)	−11.3 (4.7)	0.4 (0.2)	19.5 (4.7)	19.9 (4.9)	−19.1 (4.6)
			10 s		4940.8 (67.8)	3113.8 (1764.0)	2.5 (0.2)	1.6 (0.9)	0.8 (0.008)	0.7 (0.09)	1.8 (6.1)_	25.1 (27.0)	26.9 (20.9)	−23.3 (33.0)	3.7 (1.9)	59.6 (14.8)	63.3 (16.5)	−55.9 (13.1)
			60 s		1267.3 (78.4)	596.8 (542.3)	3.9 (0.2)	1.8 (1.6)	0.9 (0.008)	0.8 (0.02)	10.1 (35.7)	75.9 (153.9)	86.0 (118.2)	−65.8 (189.6)	20.9 (11.1)	222.1 (43.9)	243.0 (54.8)	−201.2 (33.2)

**Table A3:** Results of activity classification for the commercial activity count, the discrete and the continuous method.

Participant-ID	Project	Participant	Total Time (min)	Total Time	% in Light			% in Moderate			% in Vigorous			% in Very Vigorous		
					Commercial Activity Counts	Matlab Discrete Method	Matlab Continuous Method	Commercial Activity Counts	Matlab Discrete Method	Matlab Continuous Method	Commercial Activity Counts	Matlab Discrete Method	Matlab Continuous Method	Commercial Activity Counts	Matlab Discrete Method	Matlab Continuous Method
1	Activities of daily living—24-h data collection	Subject 1	1440	1D 0H 0M 0S	55.97	55.97	54.59	22.92	22.92	39.00	13.06	13.06	5.13	8.06	8.06	1.28
2		Subject 2	1440	1D 0H 0M 0S	65.00	65.00	55.06	16.81	16.81	37.86	12.43	12.43	4.79	5.76	5.76	2.30
3		Subject 3	1440	1D 0H 0M 0S	86.60	86.60	94.76	11.18	11.18	5.00	2.01	2.01	0.05	0.21	0.21	0.19
4		Subject 4	1440	1D 0H 0M 0S	72.50	72.50	75.08	10.00	10.00	17.75	5.49	5.49	6.45	12.01	12.01	0.72
		Mean (SD)			70.0 (13.0)	70.0 (13.0)	69.87 (19.1)	15.23 (6.0)	15.23 (6.0)	24.90 (16.5)	8.25 (5.4)	8.25 (5.4)	4.10 (2.8)	6.51 (4.9)	6.51 (4.9)	1.12 (0.9)
5	Bilateral manipulation in daily living tasks	Subject 1 (dom)	12	12M 0S	16.67	16.67	17.04	25.00	25.00	63.96	58.33	58.33	19.00	0.00	0.00	0.00
6		Subject 1 (non-dom)	11	11M 0S	9.09	9.09	5.47	36.36	36.36	76.63	54.55	54.55	17.90	0.00	0.00	0.00
7		Subject 2 (dom)	12	12M 0S	25.00	25.00	32.92	41.67	41.67	62.36	33.33	33.33	4.72	0.00	0.00	0.00
8		Subject 2 (non-dom)	11	11M 0S	18.18	18.18	21.95	27.27	27.27	73.26	45.45	45.45	4.80	9.09	9.09	0.00
9		Subject 3 (dom)	67	1H 7M 0S	62.69	62.69	31.17	20.90	22.39	56.62	14.93	13.43	12.21	1.49	1.49	0.00
10		Subject 3 (non-dom)	67	1H 7M 0S	59.70	59.70	29.60	22.39	22.39	54.55	16.42	16.42	15.85	1.49	1.49	0.00
11		Subject 4 (dom)	74	1H 14M 0S	68.92	68.92	68.73	22.97	22.97	29.75	8.11	8.11	1.52	0.00	0.00	0.00
12		Subject 4 (non-dom)	74	1H 14M 0S	62.16	62.16	62.26	29.73	29.73	34.17	8.11	8.11	3.57	0.00	0.00	0.00
13		Subject 5 (dom)	63	1H 3M 0S	49.21	49.21	4.07	25.40	25.40	75.14	22.22	22.22	20.79	3.17	3.17	0.00
14		Subject 5 (non-dom)	62	1H 2M 0S	48.39	48.39	3.58	20.97	20.97	63.13	29.03	29.03	33.29	1.61	1.61	0.00
15		Subject 6 (dom)	10	10M 0S	10.00	10.00	12.76	0.00	0.00	14.70	70.00	70.00	71.57	20.00	20.00	0.97
16		Subject 6 (non-dom)	9	9M 0S	0.00	0.00	3.04	33.33	33.33	25.00	55.56	55.56	71.11	11.11	11.11	0.84
17		Subject 7 (dom)	11	11M 0S	27.27	27.27	13.03	9.09	9.09	77.12	54.55	54.55	9.85	9.09	9.09	0.00
18		Subject 7 (non-dom)	10	10M 0S	10.00	10.00	4.75	50.00	50.00	81.62	30.00	30.00	13.63	10.00	10.00	0.00
19		Subject 8 (dom)	14	14M 0S	0.00	0.00	2.37	35.71	35.71	21.35	50.00	50.00	76.28	14.29	14.29	0.00
20		Subject 8 (non-dom)	14	14M 0S	0.00	0.00	6.64	42.86	42.86	41.24	50.00	50.00	52.12	7.14	7.14	0.00
21		Subject 9 (dom)	63	1H 3M 0S	61.90	61.90	33.36	26.98	26.98	63.33	11.11	11.11	3.31	0.00	0.00	0.00
22		Subject 9 (non-dom)	63	1H 3M 0S	63.49	63.49	37.25	26.98	26.98	62.30	9.52	9.52	0.45	0.00	0.00	0.00
23		Subject 10 (dom)	64	1H 4M 0S	59.38	59.38	27.52	26.56	26.56	58.12	9.38	9.38	14.36	4.69	4.69	
24		Subject 10 (non-dom)	65	1H 5M 0S	66.15	66.15	43.83	24.62	24.62	46.57	7.69	7.69	9.60	1.54	1.54	0.00
25		Subject 11 (dom)	64	1H 4M 0S	57.81	57.81	3.17	20.31	20.31	69.81	10.94	10.94	25.74	10.94	10.94	1.27
26		Subject 11 (non-dom)	63	1H 3M 0S	58.73	58.73	0.79	11.11	11.11	55.38	14.29	14.29	43.83	15.87	15.87	0.00
		Mean (SD)			37.9 (25.8)	37.9 (25.8)	21.2 (19.7)	26.4 (11.3)	26.4 (11.3)	54.8 (19.5)	30.2 (20.8)	30.1 (20.8)	23.9 (23.9)	5.5 (6.1)	5.5 (6.1)	0.1 (0.4)
27	Manual wheelchair propulsion	Subject 1 (day 1)	18,751	13D 0H 31M 0S	96.78	96.78	99.68	3.17	3.17	0.31	0.04	0.04	0.01	0.01	0.01	0.00
28		Subject 1 (day 2)	41,371	28D 17H 31M 0S	98.86	98.86	99.76	1.12	1.12	0.24	0.02	0.02	0.00	0.00	0.00	0.00
29		Subject 2 (day 1)	17,408	12D 2H 8M 0S	97.60	97.60	99.27	2.15	2.15	0.70	0.24	0.24	0.03	0.01	0.01	0.00
30		Subject 2 (day 2)	Missing data													
31		Subject 3 (day 1)	12,712	8D 19H 52M 0S	96.64	96.64	99.29	3.29	3.29	0.71	0.07	0.07	0.00	0.00	0.00	0.00
32		Subject 3 (day 2)	17,618	12D 5H 38M 0S	96.04	96.04	99.12	3.84	3.84	0.88	0.11	0.11	0.00	0.00	0.00	0.00
33		Subject 4 (day 1)	15,836	10D 23H 56M 0S	93.97	93.97	99.75	5.93	5.93	0.25	0.10	0.10	0.00	0.00	0.00	0.00
34		Subject 4 (day 2)	40,495	28D 2H 55M 0S	96.69	96.69	99.84	3.29	3.29	0.16	0.02	0.02	0.00	0.00	0.00	0.00
35		Subject 5 (day 1)	13,756	9D 13H 16M 0S	96.03	96.03	99.52	3.79	3.79	0.37	0.18	0.18	0.11	0.00	0.00	0.00
36		Subject 5 (day 2)	22,635	15D 17H 15M 0S	95.04	95.04	98.98	4.79	4.79	0.96	0.14	0.14	0.06	0.03	0.03	0.00
37		Subject 6 (day 1)	34,020	23D 15H 0M 0S	99.44	99.44	99.90	0.52	0.52	0.08	0.04	0.04	0.01	0.00	0.00	0.00
38		Subject 6 (day 2)	17,185	11D 22H 25M 0S	98.57	98.57	99.71	1.36	1.36	0.29	0.07	0.07	0.00	0.01	0.01	0.00
39		Subject 7 (day 1)	17,280	12D 0H 0M 0S	98.37	98.37	99.79	1.52	1.52	0.21	0.09	0.09	0.00	0.01	0.01	0.00
40		Subject 7 (day 2)	9916	6D 21H 16M 0S	97.90	97.90	99.76	1.97	1.97	0.24	0.13	0.13	0.00	0.00	0.00	0.00
		Mean (SD)			97.1 (1.6)	97.1 (1.6)	99.6 (0.3)	2.8 (1.6)	2.8 (1.6)	0.4 (0.3)	0.1 (0.06)	0.1 (0.06)	0.02 (0.03)	0.01 (0.008)	0.0049 (0.008)	0.0002 (0.0004)
41	Real-world working environment—Nordic expedition project on the Amundsen	Subject 1 (leg)	32,025	22D 5H 45M 0S	94.56	94.56	99.13	4.99	4.99	0.46	0.38	0.38	0.38	0.07	0.07	0.03
42		Subject 1 (trunk)	33,529	23D 6H 49M 0S	97.43	97.43	99.46	2.16	2.16	0.13	0.15	0.15	0.24	0.26	0.26	0.16
43		Subject 2 (leg)	34,883	24D 5H 23M 0S	94.46	94.46	99.32	5.44	5.44	0.64	0.09	0.09	0.03	0.02	0.02	0.00
44		Subject 2 (trunk)	30,109	20D 21H 49M 0S	96.33	96.33	99.40	3.56	3.56	0.50	0.08	0.08	0.07	0.04	0.04	0.03
		Mean (SD)			95.7 (1.4)	95.7 (1.4)	99.3 (0.1)	4.0 (1.5)	4.0 (1.5)	0.4 (0.2)_	0.2 (0.1)	0.2 (0.1)	0.2 (0.2)	0.1 (0.1)	0.1 (0.1_	0.1 (0.1)
